# Genomic Clustering and Homology between HET-S and the NWD2 STAND Protein in Various Fungal Genomes

**DOI:** 10.1371/journal.pone.0034854

**Published:** 2012-04-06

**Authors:** Asen Daskalov, Mathieu Paoletti, Frédérique Ness, Sven J. Saupe

**Affiliations:** Institut de Biochimie et de Génétique Cellulaire, UMR 5095, CNRS - Université de Bordeaux 2, Bordeaux, France; University of California Riverside, United States of America

## Abstract

**Background:**

Prions are infectious proteins propagating as self-perpetuating amyloid polymers. The [Het-s] prion of *Podospora anserina* is involved in a cell death process associated with non-self recognition. The prion forming domain (PFD) of HET-s adopts a β-solenoid amyloid structure characterized by the two fold repetition of an elementary triangular motif. [Het-s] induces cell death when interacting with HET-S, an allelic variant of HET-s. When templated by [Het-s], HET-S undergoes a trans-conformation, relocates to the cell membrane and induces toxicity.

**Methodology/Principal Findings:**

Here, comparing HET-s homologs from different species, we devise a consensus for the HET-s elementary triangular motif. We use this motif to screen genomic databases and find a match to the N-terminus of NWD2, a STAND protein, encoded by the gene immediately adjacent to *het-S*. STAND proteins are signal transducing ATPases which undergo ligand-induced oligomerisation. Homology modelling predicts that the NWD2 N-terminal region adopts a HET-s-like fold. We propose that upon NWD2 oligomerisation, these N-terminal extensions adopt the β-solenoid fold and template HET-S to adopt the amyloid fold and trigger toxicity. We extend this model to a putative prion, the σ infectious element in *Nectria haematococca,* because the *s* locus controlling propagation of σ also encodes a STAND protein and displays analogous features. Comparative genomic analyses indicate evolutionary conservation of these STAND/prion-like gene pairs, identify a number of novel prion candidates and define, in addition to the HET-s PFD motif, two distinct, novel putative PFD-like motifs.

**Conclusions/Significance:**

We suggest the existence, in the fungal kingdom, of a widespread and evolutionarily conserved mode of signal transduction based on the transmission of an amyloid-fold from a NOD-like STAND receptor protein to an effector protein.

## Introduction

Prions are infectious proteins that propagate information embedded in their conformational state. Prions are responsible for fatal neurodegenerative diseases such as CJD and kuru in humans, BSE in cattle, Scrapie in sheep and CWD in cervids [1], [2]. In these infectious diseases, the host PrP protein misfolds into self-perpetuating amyloid aggregates. Prions have also been identified in fungi, initially as non-Mendelian genetic elements [3], [4]. These proteins exist in a soluble conformation and in an infectious amyloid conformation. Nine prion proteins have been identified in the yeast *Saccharomyces cerevisiae* [5] and one in the filamentous fungus *P. anserina* [6]. Prion formation most often corresponds to an inactivation of the protein which is no longer capable of performing its cellular function because it is trapped in amyloid aggregates. The biological significance of the prion phenomenon in fungi is still highly debated [4]. Some view yeast prions as infectious protein misfolding diseases, the conceptual counterpart of prion diseases in mammals [7], [8], [9]. Others advocate that prion formation can sometimes represent an adaptive mechanism, allowing for an epigenetic regulation of protein activity [10], [11], [12], [13].

The HET-s prion protein of the filamentous fungus *P. anserina* is involved in a non-self recognition process. [14], [15]. In filamentous fungi, when cell fusions between different individuals occur the mixed cells are destroyed by a cell death reaction [16]. This reaction is termed heterokaryon incompatibility and is controlled by specific genes termed *het* genes. Classically, it is proposed that incompatibility serves to limit the transmission of mycoviruses and other deleterious replicons between strains. The *het-s/het-S* genes constitute one of many gene pairs controlling heterokaryon incompatibility. A fusion between a *het-s* and a *het-S* strain is lethal. The protein encoded by the *het-s* gene is a prion and exists under a soluble form and an aggregated self-propagating prion form. *Het-s* strains acquire the prion state either spontaneously at a low frequency or systematically after a cell fusion with a prion-infected strain.

**Figure 1:**
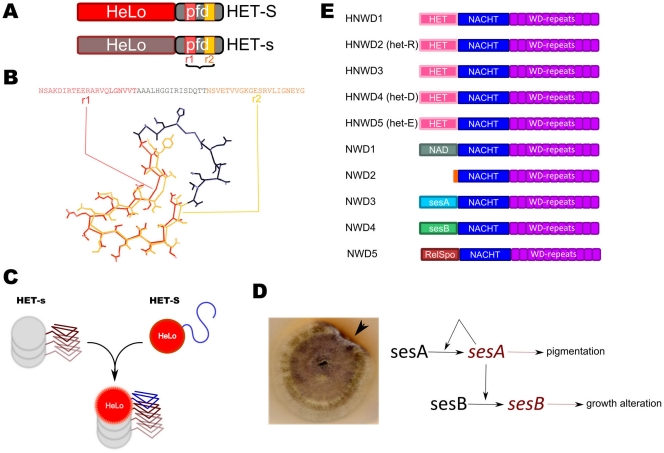
Introductory figure. A. Diagram of the HET-s and HET-S domain organization with a N-terminal HeLo domain and a C-terminal PFD comprising two pseudo-repeats (r1 and r2). **B.** Structure of the HET-s PFD (pdb: 2RNM), only the two pseudo-repeats and the connecting loop are shown. **C.** Mechanism of HET-S/[Het-s] incompatibility, when the HET-S PFD region interacts with the prion form of HET-s, transconformation of the C-terminal region induces refolding and activation of the HeLo toxicity domain. **D.** The “secteur” phenotype caused by the σ infectious element in *Nectria haematococca.* The arrow points to a mycelia sector in which the σ infectious element is present and leads to an alteration of the growth margin and to the secretion of a red pigment. The diagram on the right is the working model proposed by Daboussi and co-workers to account for the role of the sesA and sesB proteins. The model proposes that sesA and sesB can exist in a normal and modified form (italicized) and that the modification of sesA is autocatalytic and can also induce modification of sesB. Modified sesA leads to the red pigmentation phenotype, modification of sesB to the growth alteration characteristic of the “secteur” (redrawn after Graziani et al. 2004). **D.** Domain organization of the members of NWD-gene family of *P. anserina*. All family members share a central NACHT nucleotide binding oligomerisation domain and a C-terminal WD repeat domain but differ by their N-terminal effector domains. This effector domain is a HET cell death inducing domain (PF06985) in NWD1 to NWD5 (see text for description of the other effector domains). NWD2 lacks a N-terminal effector domain.

The activity in heterokaryon incompatibility is associated with the prion form of HET-s [15], [17], [18]. Indeed, incompatibility is triggered when the prion form of HET-s interacts with the HET-S allelic variant. HET-s and HET-S are both 289 amino acids in length and differ by 13 residues. HET-s and HET-S are two-domain proteins with a C-terminal prion forming domain (PFD, residue 218 to 289) necessary and sufficient for prion propagation and a N-terminal α-helical globular domain (residue 1-∼227) designated HeLo domain that partially overlaps with the PFD [19], [20], [21], (Figure 1A). The PFD is natively unfolded in the soluble form of the protein and -upon prion formation- undergoes a transition to a β-sheet structure. The structure of HET-s PFD has been solved by solid state NMR, making HET-s currently the only prion model for which a high resolution structure is available [22], [23]. A 8.5 Å resolution cryo-electron microscopy reconstitution of the HET-s fibrils has also been reported [24]. The PFD is composed of two repeated motifs of 21 amino acids connected by a large flexible loop of 15 amino acids (Figure 1B) and adopts a β-solenoid structure with two layers of β-strands per monomer, each layer being composed of one of the repeated motifs [23], [24], [25], [26]. The β-strands delimit a triangular hydrophobic core with an additional fourth β-strand protruding from the core and delimiting a pocket filled by a C-terminal loop containing aromatic residues [23], [27]. The HET-s and HET-S PFD regions are functionally equivalent and interchangeable; the functional difference between HET-s and HET-S are determined by the amino acid differences in their HeLo domains (in particular position 33) [19], [28]. The HeLo domain of HET-S represents the cell death execution domain in the *het-s/het-S* system [19], [20], [29]. It is proposed that in the incompatibility reaction, when the PFD region of HET-S interacts with the prion form of HET-s, the conversion of the HET-S PFD region to the β-solenoid fold induces a conformational change in the HeLo domain leading to its activation and triggering cell death by incompatibility [20], (Figure 1A). It was recently shown that upon interaction with [Het-s], HET-S relocates to the cell periphery and that this cell periphery localisation correlates with cell death [30]. In contrast, HeLo domain of HET-s does not exert any toxicity.

A number of HET-s orthologs have been identified in other pezizomycota and one of them, FgHet-s, from the plant pathogen *Fusarium graminearum,* was structurally and functionally characterized [31]–[32]. FgHET-s(218–289), the C-terminal domain of the *F. graminearum* protein forms amyloids and behaves as a prion *in vivo* in heterologous hosts like yeast or *P. anserina*. FgHET-s(218–289) cross-seeds HET-s(218–289) and *vice versa* [31]. A structure model of FgHET-s(218–289) based on hydrogen exchange and solid state NMR predicts a β-helical amyloid fold with a triangular hydrophobic core very similar to the one characterized for HET-s [31]. This retention of the amyloid forming ability occurred in spite of extensive sequence divergence (the two domains show only 38% identity) but with conservation of key residues, critical for the β-solenoid fold. There is thus evidence for the existence of a selective pressure for the maintenance of the ability to form that specific amyloid fold in fungi [31]–[32].

So far, no other prions than [Het-s] have been formally identified in filamentous fungi. But, the existence of a prion system in the species *Nectria haematococca* is strongly suspected. The “secteur” phenotype of *N. haematococca* corresponds to a growth alteration and dark red pigmentation of the mycelium at the growth margin of the colony [33], (Figure 1C). The “secteur” phenotype appears spontaneously at a low frequency, is transmissible by cytoplasmic contact and does not involve a genetic modification (in the classical sense). The “secteur” phenotype is due the formation and transmission of a cytoplasmic determinant (designated “σ”). σ can be reversibly cured and the formation and propagation of σ are dependent on a nuclear genetic locus called *s*. Certain mutations at the *s* locus can abolish σ formation while others make σ constitutive. Molecular cloning of the *s* locus was achieved and it was found to comprise two genes termed *sesA* and *sesB*. *sesA* encodes a protein of unknown function and *sesB* encodes a protein with a α/β hydrolase fold. Daboussi and co-workers proposed that σ corresponds to a modified form of the sesA and sesB proteins and that modified sesA is able to promote modification of sesB [33], (Figure 1C). The modified form of sesA would lead to red pigmentation and modified sesB would cause the growth alteration. An hypothesis envisioning σ as a prion of sesA and sesB is fully compatible with the current data but awaits confirmation.

Signal transducing NTPases of the STAND type play essential roles in the regulation of programmed cell death and immune response in plants, animals and fungi [34] and several *het* incompatibility genes of *P. anserina* encode STAND proteins [35], [36], [37], [38]. STAND proteins typically associate a N-terminal effector domain to a central nucleotide binding oligomerisation domain (NOD) of the NACHT or NB-ARC type and a C-terminal ligand-binding domain composed of superstructure-forming repeats such as WD, LRR, TPR or ANK repeats. The mechanism of ligand-induced oligomerisation of STAND proteins is best understood for APAF-1 and its fly and worm homolog [39]–[41] [42], [43] [44], which thus serve as paradigms for other STAND-proteins [45], [46] [47]. APAF-1 controls the activation of the initiation caspase-9 in the intrinsic apoptosis pathway. APAF-1 displays a N-terminal CARD effector domain, followed by a NOD domain and C-terminal WD-repeat domain. Upon binding of cytochrome *c* to the WD-repeats, APAF-1 undergoes a transition from a monomer to an oligomeric superstructure termed apoptosome, a ring shaped heptamer in which the NOD domains form a central ring. The oligomerisation step groups the CARD domains into a hub where binding and activation of caspase-9 occurs. Other STAND proteins with immune functions, like the mammalian NOD-like receptors, also oligomerise upon ligand binding to the repeat domain [48] [45], [46] [49]. The assembled form of these proteins has been termed inflammasome and in the case of the NALP1 NOD-like receptor, a structure with a 5 or 7-fold symmetry was reported (Faustin et al. 2007). Not all STAND proteins oligomerise into monodisperse annular structures, the prokaryotic MalT transcriptional regulator, oligomerises into polydisperse curvilinear oligomers [50].

The STAND proteins of *P. anserina* involved in incompatibility (*het-E*, *het-D* and *het-R*) belong to a gene family designated NWD. This gene family comprises 10 members. All 10 members share a C-terminal WD-repeat domain and a central NACHT domain (HET-E is one of the proteins in which this domain was originally identified, NACHT is an acronym for NAIP, CIITA, HET-E, TP-1) [51], (Figure 1D). The best studied NWD protein of *P. anserina* is HET-E (HNWD5). Incompatibility is triggered when HET-E interacts with specific allelic variants of a protein termed HET-C [52]–[53]. *Het-c* encodes a GLTP (glycolipid transfer protein). GLTPs are cytosolic proteins conserved in most eukaryotes which bind specific glycolipids but their exact cellular function remains debated [54]. The WD-repeat domain of HET-E is responsible for recognition of HET-C variants [35], [36], [38]. A mutation in the P-loop, abolishing nucleotide binding, inactivates HET-E [35], [55]. Based on the APAF-1 paradigm, it is proposed that upon binding of the HET-C GLTP to the WD-repeat domain, HET-E undergoes an oligomerisation process which would lead to activation of the HET cell death effector domain [56]. Five members of the NWD-gene family, HNWD1 to HNWD5, display this same N-terminal HET cell death effector domain, while the other members show different N-terminal effector domains. NWD3 and NWD4 display N-terminal domains homologous respectively to the afore mentioned sesA and sesB proteins of *N. haematococca* [33]. The NWD5 N-terminal domain is homologous to the prokaryotic RelSpo regulator of ppGpp synthesis, the alarmone inducing the stringent response in bacteria [57]. NWD1 displays a unidentified domain we term NAD (for NWD1 associated domain). Finally, NWD2 stands out as an exception in this gene family since this protein lacks a defined N-terminal effector domain upstream of the NACHT nucleotide binding oligomerisation domain.

In the present paper, based on sequence analyses, we propose that the NWD2 STAND protein is a functional partner of the HET-S protein. We propose a model in which ligand induced oligomerisation of NWD2 leads to amyloid transconformation of HET-S. These observations impose a re-evaluation of biological significance of the [Het-s] prion system and introduce the notion that propagation of an amyloid fold can be an integral part of a signal transduction mechanism. We expand this model to another putative prion model of the fungus *N. haematococca* and identify several other potential prion-like proteins that could be controlled by STAND proteins.

## Results

### Defining a Consensus Sequence for the Elementary Repeat Unit of the β-solenoid Fold of HET-s

**Figure 2:**
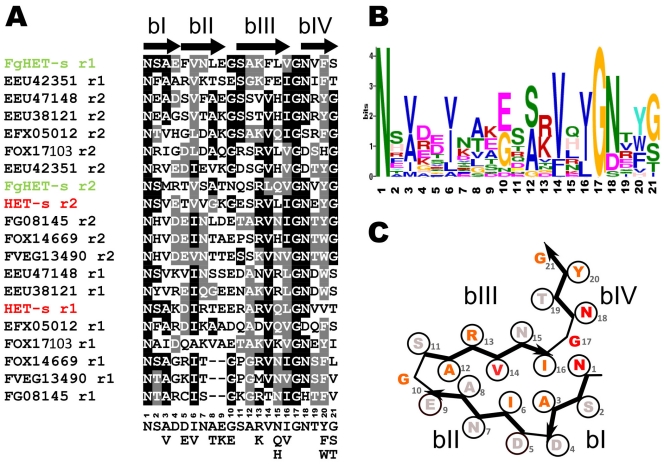
Defining a consensus sequence for the elementary unit of HET-s β-solenoid fold. **A.** alignment of the 21 amino acid repeat units of HET-s, FgHET-s and 9 other HET-s homologs from various fungal species, r1 and r2 designate the first and second repeat unit respectively. The arrows above the alignment represent the 4 β-strands defining the β-solenoid fold (bI to bIV). Below the alignment the corresponding consensus sequence is given. FOX1713 and FOX14669 are from *Fusarium oxysporum*, FG08145 is from *Fusarium graminearum*, FVEG13490 is from *Fusarium verticillioides*, EEU42351, EEU47148, EEU38121 are from *Nectria haematococca*, EFX055012 is from *Grosmannia clavigera*. **B.** Weighted consensus of the HET-s elementary repeat unit generated using the MEME algorithm. **C.** Fitting of the consensus sequence for the elementary HET-s repeat into the β-solenoid fold model. The colour coding reflects the weight of the residue in the MEME consensus (red, very strong conservation, informational content >2 bits; orange, strong conservation, >1 bit; grey, weak conservation, <0.5 bit).

HET-s homologs were identified in the genome sequences of *F. graminearum, F. oxysporum*, *F. verticillioides*, *N. haematococca* and *Grosmannia clavigera*. The sequences of the PFD region of 9 HET-s homologs were aligned with ClustalW and the sequence stretch matching the elementary r1 and r2 repeat motifs of HET-s were extracted. The elementary repeat motifs where then aligned anew in order to generate a consensus sequence of the elementary repeat unit of the β-solenoid fold (Figure 2A). This alignment of a total of 20 elementary motifs leads to a 21 amino acids long consensus (Figure 2B). This consensus can be fitted into the triangular hydrophobic core model of HET-s (Figure 2C). The consensus reveals the strong conservation of key residues for the formation of the β-solenoid fold, such as the asparagine ladder forming N residues in position 1 and 18, the large hydrophobic residues in the core in position 3, 6, 14 and 16 and the glycine residue in the β-arch in position 17 and to a lesser extend in position 10. Position predicted to be solvent exposed and located outside of the hydrophobic core show a preference for polar residues and charged residues (positions 2, 4, 5, 7, 9, 11, 13 and 15). Of note is the preference for a positively charged residue at position 13.

### The N-terminus of the NWD2 Protein Matches the Consensus Sequence for the Elementary HET-s and is Predicted to Adopt a HET-s-like Fold

**Figure 3:**
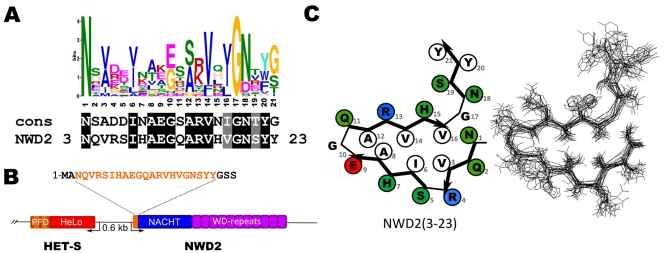
The N-terminal end of the NWD2 protein matches the HET-s PFD consensus. **A.** Comparison of the NWD2 sequence with the HET-s consensus sequence. **B.** Diagram showing the genome organization of *het-s* and *nwd2* as two divergently transcribed adjacent genes. **C.** Fitting of the NWD2 (3-23) sequence into the β-solenoid fold model (left) and homology model of the NWD2(3-23) sequence (right). Polar residues are given in green, hydrophobic residues are given in white and positively and negatively charged residues in blue and red, respectively.

A search for sequences matching the consensus defined above in the non-redundant NCBI protein database was performed using the PSI-Blast algorithm. In this search, the second best match was found to the *P. anserina* NWD2 protein (Figure 3A), (the best match was to the *F. graminearum* FG8145 protein, one of the HET-s homologs used to generate the consensus). The *nwd2* gene is located immediately adjacent to the *het-s/het-S* gene in the *P. anserina* genome (Figure 3B). The sequence matching the consensus corresponds to the N-terminus of the NWD2 protein (residue 3 to 23) (Figure 3B). The 21 amino acid motif is found only once in NWD2 and not repeated twice as in the case of the HET-s PFD. Based on homology modeling, the NWD2(3-23) region is predicted to adopt a HET-s-like fold with formation of a triangular hydrophobic core (Figure 3C). In this homology model, polar residues locate outside of the core, bulky hydrophobic residues in the inside with smaller (alanine) residues close to the β-arch (position 8 and 12). The two glycines in the β-arch regions (position 10 and 17) are present, as well as the asparagines ladder forming N residues (positions 1 and 18). There is a positive charge in position 13. As mentioned previously, NWD2 protein is a member of the NWD gene family (Figure 1D) and lacks a defined N-terminal effector domain upstream of the NACHT domain but in place bears this region of homology to the HET-s PFD motif (Figure 3C).

Of note here is the fact that *het-s* and *het-S* wild-type strain are also polymorphic for *nwd2*. In all wild isolates of the *het-s* genotype analyzed so far, the *nwd2* gene is inactivated by insertion of a transposon, the REPA repetitive element [28], [58]. In other words, only *het-S* strains harbor a functional *nwd2* gene while the gene is inactivated in *het-s* strains.

### Evolutionary Conservation of the HET-S/STAND Clustering and Homology in other Fungal Species

We find a clustering and sequence homology between *het-s* and a STAND-encoding gene in *P. anserina*. We next asked whether these features are evolutionarily conserved in other fungal species. We have thus analyzed the genome organization around the *het-S*-homologs in different species.

**Figure 4:**
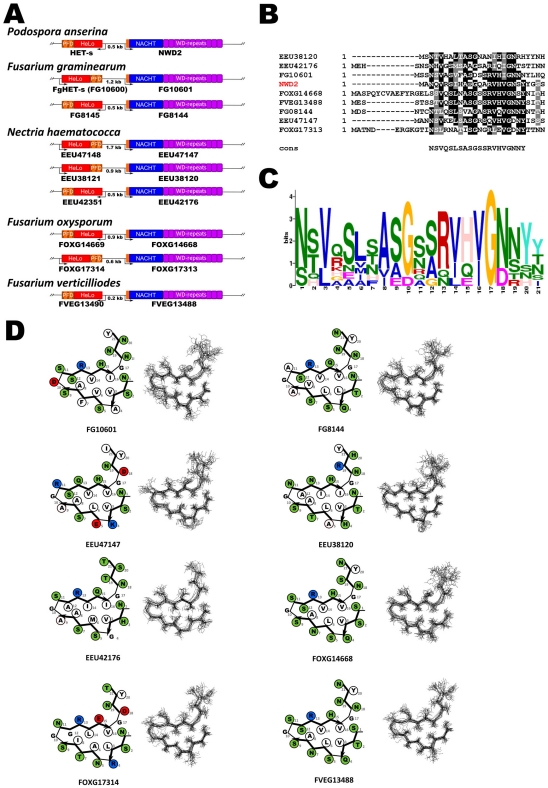
Evolutionary conservation of the *nwd2/het-S* genomic association and homology. A. Genomic organization of the het-s homologs found in various filamentous fungi. Note that in all cases the *het-S* and *nwd2* homologs are encoded by adjacent genes but gene orientation differs between species. **B.** Sequence alignment of the N-terminal end of the NWD2-homologs from various species. **C.** Weighted consensus based on the alignment in Figure 4B and generated with the MEME algorithm. **D.** Fitting of the N-terminal sequences of the NWD2-homologs into the β-solenoid fold model (left) and homology model of the same sequences (right), polar residues or given in green, hydrophobic residues are given in white and positively and negatively charged residues in blue and red, respectively.

We found that in all analyzed species, the genes encoding the HET-S homologs are immediately adjacent to genes encoding STAND proteins (Figure 4A). In certain gene pairs, the genes are divergently transcribed as seen in *P. anserina,* while in others the gene are transcribed in the same direction (Figure 4A). In all cases these STAND proteins display a N-terminal sequence homologous to the elementary HET-s PFD motif (Figure 4B). Residues conserved between the different STAND homologs correspond to the highly conserved residues in the HET-s β-solenoid fold consensus (Figure 4B). The weighted consensus of the N-terminal extensions of the HET-s associated STAND proteins (Figure 4C), closely resembles the HET-s elementary repeat consensus. Again conservation of the N in position 1 and 18, of the G in position 10 and 17 and hydrophobic residues in position 3, 6, 14 and 16 are apparent. Here also, there is conservation of a positively charged R residue in position 13. Positions variable in the HET-s repeat consensus are also more variable in the alignment of the N-terminal sequences of the STAND proteins. In all cases, the N-terminal sequences of the STAND proteins are predicted by homology modeling to be able to adopt a HET-s-like fold (Figure 4D).

We find that the *het-s* locus is immediately adjacent to a gene encoding a STAND protein which displays a N-terminal sequence homologous to the HET-s β-solenoid fold consensus. This NWD2 protein is part of a gene family comprising several members involved in heterokaryon incompatibility. This STAND protein lacks an N-terminal effector domain in contrast to the other members of the gene family. These features are widely evolutionarily conserved in all analyzed *het-s* homologs.

**Figure 5:**
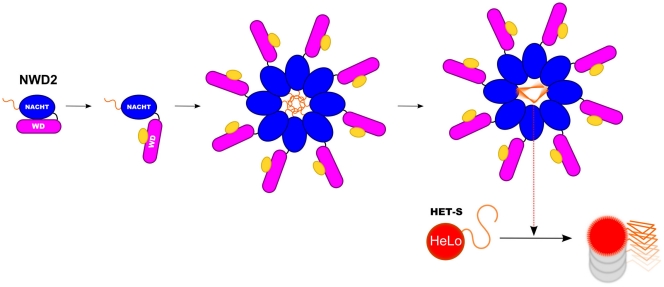
Proposed model for the NWD2/HET-S interaction. Based on the APAF-1 paradigm for STAND protein activation mechanism, it is proposed that NWD2 exists in an inactive closed state in the absence of its cognate ligand and undergoes a transition to an open state upon ligand binding to the WD-repeat region, which allows for oligomerization via the NOD domains. This oligomerization step is proposed to bring the N-terminal extensions of NWD2 molecules into close proximity and to allow their cooperative folding into the β-solenoid fold. In this model, this amyloid-like fold then serves as a template to nucleate HET-S transconformation and activation of the HeLo toxicity domain.

We propose a model postulating the existence of a functional interaction between NWD2 and HET-S that can account for these observations (Figure 5). Based on the APAF-1 paradigm, we propose that NWD2 can recognize a ligand via its WD-40 repeat domain and oligomerizes in response to this binding. This oligomerization step would put the N-terminal extensions of NWD2 proteins into close proximity and allow their cooperative folding into the β-solenoid fold. Once formed this fold would be used as a template for transconformation of the HET-S PFD. This transconformation triggers activation of the HeLo toxicity domain [20].

### HeLo-domain Proteins with a PFD-like Region Distinct from the HET-s Motif

**Figure 6:**
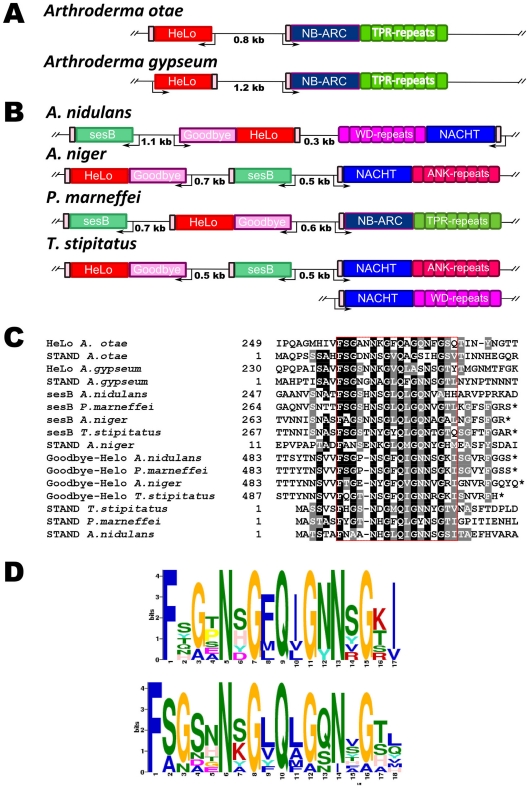
Distant HET-S homologs are also associated to genes encoding STAND proteins. **A.** Genome organization of two distant HET-S homologs with a HeLo domain from Arthroderma species. The pink box depicts the region of homology between the C-terminal region of the HET-S homolog and the N-terminal region of the STAND protein. **B.** Genome organization HET-S homologs with a HeLo domain and Goodbye domain in various species. The pink box depicts the region of homology between the C-terminal region of the HET-s homolog and sesB homologs the N-terminal region of the STAND protein. **C.** Alignment of the HeLo, HeLo/Goodbye, sesB and STAND proteins depicted in A and B. **D.** Weighted consensus based on the alignment in Figure 6C and generated with the MEME algorithm.

Next, we have analyzed more distant HET-S homologs that are not picked up in a PSI-BLAST search when the HET-s PFD sequence is used as query but are only detected if the search is performed with the HeLo domain. In the genomes of dermatophyte species *Arthroderma otae* and *Arthroderma gypseum,* we identified two other HET-S-homologs. These two proteins display a HeLo domain and short sequence extension C-terminal to the HeLo domain which is distinct from to the HET-s PFD motif (see below). These two *A. otae* and the *A. gypseum* genes are also located adjacent to genes encoding STAND proteins. These STAND proteins display a N-terminal extension homologous to the extension found at the C-terminus of the HeLo domains (Figure 6A). In the present case, the STAND proteins display a TPR repeat domain in place of the WD-repeat domain found in NWD2. In addition, the NOD domain of these STAND proteins is of the NB-ARC type instead of the NACHT type found in NWD2.

We also identify 4 proteins in which the HeLo domain is part of a larger protein and associated to a second distinct domain (we designated Goodbye). In these 4 homologous proteins, this Goodbye domain is N-terminal to the HeLo domain. These proteins also display a short extension C-terminal to the HeLo domain. Genome sequence analyses revealed that the corresponding genes are all adjacent to genes encoding STAND proteins (Figure 6B). These STAND proteins all display a N-terminal sequence which shows homology to the C-terminal extension of the Goodbye-HeLo protein and to the extension of the HeLo/STAND pairs from *A. otae* and *A gypseum* described above (Figure 6B). In addition, in the immediate vicinity of the gene encoding the HeLo-Goodbye protein, is a gene encoding a sesB homolog, a gene involved in the propagation of the σ infectious element of *N. haematococca*. These sesB homologs also display the C-terminal extension homologous to the HeLo-Goodbye protein C-terminus and the STAND protein N-terminus. An alignment of the terminal extensions of this ensemble of proteins (HeLo, Goodbye-Helo, STAND and sesB) was performed and led to a consensus motif (Figure 6C). This consensus can be expressed as the 17/18 amino acid motif: FxGx(x)NxGLQLxGxNxGxL. This pattern has a pseudopalindromic structure centered on the Q residue. We designate this extension PP-motif (for pseudopalindrom).

### Expansion of the Model to the σ Infectious Element of *Nectria Haematococca*

**Figure 7:**
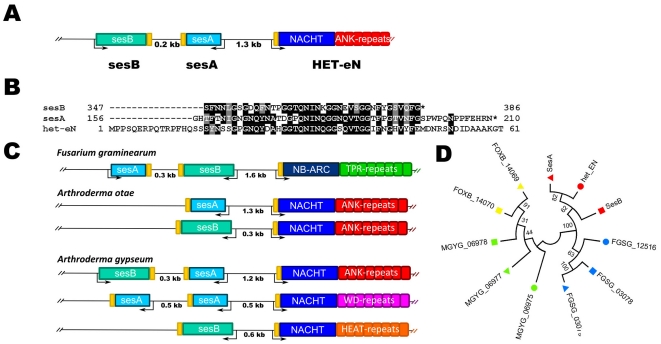
The *s* locus controlling propagation of the σ infectious element of *Nectria haematococca*. **A.** Genome organization of the *sesA* and *sesB* genes controlling formation of the σ element. Note that *sesA* and *sesB* are adjacent to the *het-eN* gene encoding a STAND protein. **B.** Sequence alignment of the C-terminal regions of sesA and sesB and the N-terminal region of HET-eN. **C.** Genome organization of the sesA and sesB loci in different fungal species. Note that sesA and/or sesB homologs are found adjacent to a gene encoding a STAND protein in various fungal species. Gene order varies from species to species. **D.** Phylogenetic tree of the sesA (triangle), sesB (square) and STAND (circle) extension from *F. graminearum* (blue), *F. oxysporum* (yellow), *N. haematococca* (red) and *Arthroderma gypsem* (green). Note that extension group by gene cluster rather than by orthologous groups. The *F. oxysporum* STAND homolog was omitted from the analysis because gene annotation is ambiguous. The evolutionary tree was constructed using the Neighbor-Joining method. The optimal tree is shown. The analysis involved 11 amino acid sequences. All positions containing gaps and missing data were eliminated. There were a total of 32 positions in the final dataset. Evolutionary analyses were conducted in MEGA5. Bootstrap values are given for each node.

The observations made on the *het-S/nwd2* clustering and the fact that sesB homologs are found associated with HET-S homologs (Figure 6) prompted us to analyze the genome organization around the *s* locus controlling propagation of the σ infectious element in *N. haematococca* (Figure 7A). Dabouzzi and co-workers have reported that adjacent to the sesA, sesB gene pair is a gene designated *het-eN* encoding a STAND protein with a NACHT domain showing homology to the NWD proteins of *P. anserina* and displaying ankyrin repeats. Sequence analyses reveal that the sesA and sesB proteins share a C-terminal region of homology (Figure 7B)[33]. The sesA and sesB proteins differ in their N-terminal region, sesB is annotated as a lipase/esterase with a α/β hydrolase fold, sesA displays an N-terminal domain of unknown function. A sequence homologous to the C-terminal region of homology common to sesA and sesB is also found in the N-terminus of the HET-eN STAND protein (Figure 7A and 7B). The region of homology between HET-eN, sesA and sesB was not detected originally by Dabouzzi and co-workers because in the annoted het-eN protein an intron in the 5′ region of the gene went unnoticed. The region of homology between sesA, sesB and HET-eN is distinct from the HET-s-PFD motif and PP-motif, nonetheless it is biased in amino acid composition and rich in G, N/Q and aromatic residues (see below).

Similarly to the model proposed for HET-S/NWD2, we propose that sesA and sesB could be prion proteins sharing homologous PFDs and that prion formation of sesA and sesB might be induced by the oligomerisation of the het-eN STAND protein. Daboussi and co-workers have proposed that modification of sesA can induce modification of sesB (Figure 1C). The homology between the sesA and sesB proposed PFDs can explain how modification (prionization) of sesA could induce modification of sesB.

We then asked whether the features of the sesA/sesB genes are evolutionarily conserved. We thus searched for sesA and sesB homologs in other fungal species. sesA and sesB homologs were found in several species including *F. graminearum, F. oxysporum* and several dermatophytes such as *A. otae* and *A. gypseum*. The sesA and sesB homologs were found adjacent to STAND protein encoding genes (Figure 7C). Gene order and orientation varied in the different species and the ses-associated STAND proteins showed a diversity of repeat types (ANK, TPR, WD or HEAT repeats) and NOD domain type (NB-ARC or NACHT). sesA and sesB clustering was not systematic, in certain loci either sesA or sesB were missing. Also, in certain loci two adjacent copies of sesA are found. When the sequences of PFD-like extension of the sesA/sesB/STAND pairs or triads from different species were compared, we found that the extensions were more similar within a gene cluster than between orthologs (Figure 7D), suggesting co-evolution of the extensions within a gene cluster.

**Figure 8:**
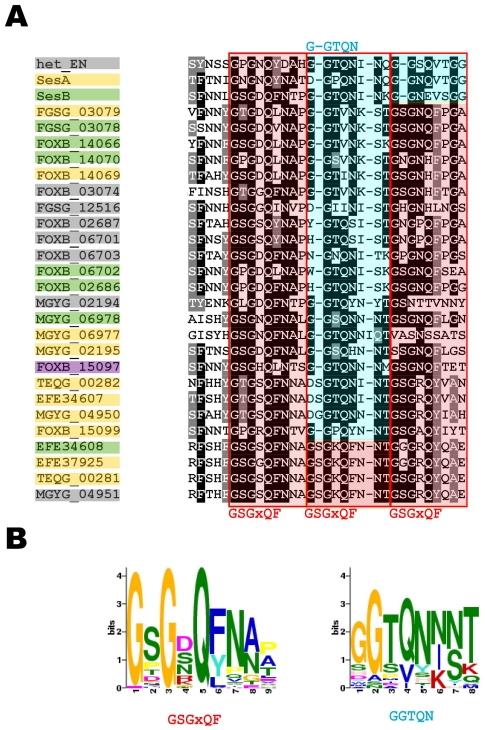
Alignment of sesA, sesB and het-eN homologs from various species and definition of the consensus σ-motif. **A.** PFD-like extension of STAND, sesA and sesB homologs (boxed in grey, yellow and green respectively; the sequence with a purple box is a NAD domain protein) from various species have been aligned using ClustalW. The A (GSGxQF) and B (GGTQN) motifs have been boxed in red and blue respectively. **B.** Weighted consensus of the A and B motifs generated with the MEME algorithm.

In order to derive a consensus sequence for the putative prion forming domains of the *N. haematococca* sesA/sesB type, we performed an alignment of the effector domain and STAND protein extensions we have identified (Figure 8A). We find that these domains comprise two motifs: motif A, GxGxQFNxx and motif B, GGTQNINT (Figure 8B). These two motifs are repeated three times in different patterns (AAA, ABA or ABB). We designate this organization, σ-motif.

### Co-existence of Two Alternate STAND Protein Architectures

**Figure 9:**
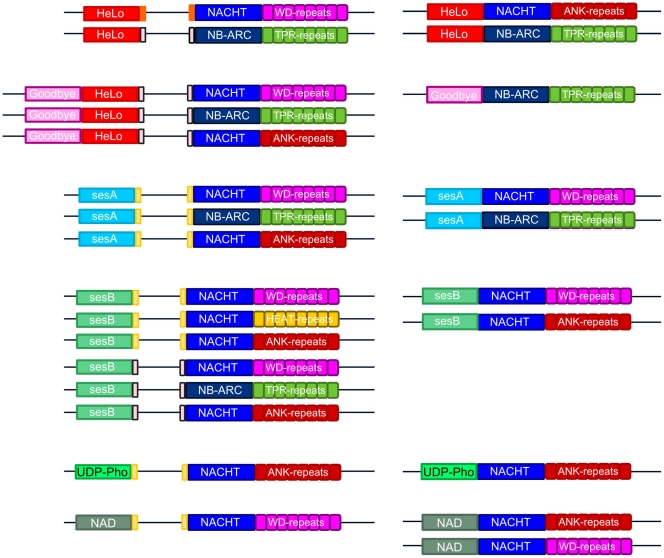
Alternate gene architectures of effector domain/STAND associations. The figure gives a side-by-side comparison of the alternate gene architecture of fungal STAND prioteins. The genes encoding the effector domain described here can be found adjacent to genes encoding STAND proteins and alternatively associated directly at the N-terminus of the STAND protein. The type of superstructure forming repeats is also variable. The HET-s motif, PP-motif and σ-motif PFD or PFD-like extension are represented as orange, pink and yellow boxes respectively.

We find that the genes encoding HeLo, sesA, sesB and Goodbye domains are associated to PFD (or PFD-like) motifs and clustered with genes encoding STAND proteins lacking effector domains. When we analyzed fungal genomes for the presence of these 4 domains in other protein architectures, we found that these same 4 domains can also been found as N-terminal effector domains of STAND proteins with a NACHT or NB-ARC NOD domain and various superstructure forming repeats (Figure 9). For instance, we have identified STAND proteins displaying a N-terminal HeLo domain either associated with a NACHT and WD-repeat domains or NB-ARC and TPR repeat domains (Figure 9). Similarly, the *P. anserina* NWD3 and NWD4 proteins represent examples of STAND proteins displaying a sesA and sesB-like N-terminal domain respectively (Figure 1D). It thus appears that the proposed STAND/effector protein couples can exist alternatively in an adjacent gene pair or in an “all-in-one” architecture. Figure 9 gives representative examples of this type of alternate domain organisations in fungal STAND proteins.

### Searching for Additional Putative Fungal STAND/Effector Domain Couples

**Figure 10:**
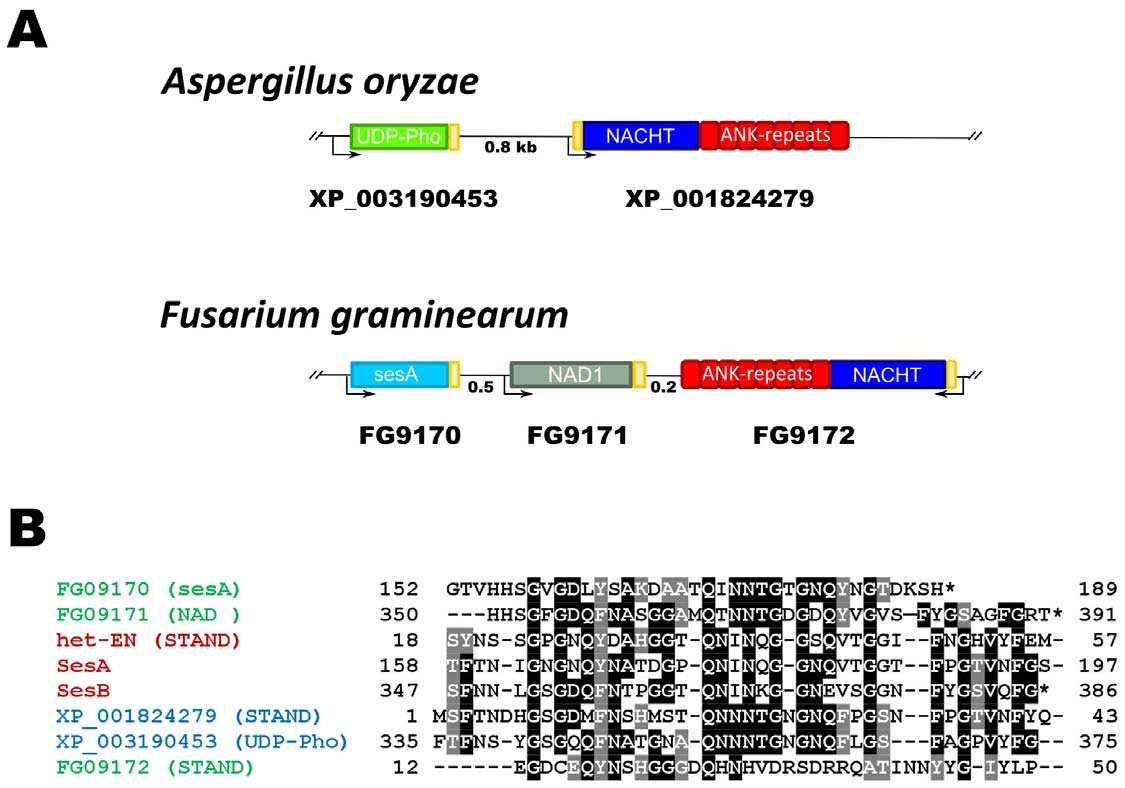
Identification of additional prion candidates. A. In *A. oryzae*, a gene encoding a protein with a UDP-Phosphorylase domain lies adjacent to a gene encoding a STAND protein and there is a region of homology between the C-terminus of that protein and the N-terminus of the STAND protein. In *F. graminearum*, a protein with a domain termed NAD lies adjacent to a gene encoding a STAND protein and adjacent to a gene encoding a sesA homolog. There is a region of homology between the C-terminus of that protein and the N-terminus of the STAND protein and the C-terminus of the sesA homolog. **B.** Alignment of the regions of homology depicted as yellow boxes in A with the sesA and sesB and het-eN proteins from *Nectria haematococca.*

Although no comprehensive catalog of fungal STAND proteins is yet available, it appears that there are multiple additional effector domains that can be found as N-terminal domains of fungal STAND proteins. We thus wondered whether additional effector domains could be part of putative STAND/prion couples in fungi. We chose a subset of 4 such STAND effector domains as candidates. We picked the HET domain (interpro: IPR010730), the RelSpo domain (interpro: IPR004811) and the NAD domain because these domains are found in genes of the NWD family. We also chose the UDP-phosphorylase domain (interpro: IPR000845) because it is among the most frequently found effector domains in fungal STAND proteins listed in the pfam or interpro directories of fungal NACHT and NB-ARC domain proteins (IPR007111 and IPR002182 respectively). We screened in Blast searches for proteins containing HET, RelSpo, NAD or UDP-phosphorylase domains as unique domains. We then looked for the presence of a gene encoding a STAND protein (lacking an effector domain) adjacent to the gene encoding the candidate domain. No RelSpo/STAND or HET/STAND pairs satisfying these search criteria were identified. In contrast, UDP-phosphorylase/STAND pairs were identified in *Aspergillus oryzae*, *Aspergillus terreus* and *Magnaporthea grisea* and NAD/STAND pairs were found in *F. graminearum* and *F. oxysporum* (Figure 9 and 10A). In this latter example, a sesA homolog is also present at the same locus. The respective C-terminal extension and N-terminal extension of the effector domains and the STAND proteins show homology to the σ-type PFD-like extension domain of the sesA/sesB genes of *N. haematococca* (Figure 10B). When, the sequences of the extensions in these proteins are compared, they tend to group by gene cluster rather than by orthologous groups as already described for the sesA/sesB/STAND triads (not shown), arguing again for co-evolution of the extensions within a cluster.

As an alternate approach, we also performed PSI-BLAST searches with the HET-s, PP- and σ-motifs in order to identify additional prion candidates. No other effector domain than the already identified HeLo, Goodbye-HeLo, sesA, sesB, NAD and UDP-phosphorylase domain proteins were identified in that way. We however identified a *N. haematococca* protein (EEU38904.1) displaying a C-terminal extension matching the σ-motif, but the gene encoding this protein was not adjacent to a STAND encoding gene.

Together, these analyses reveal that the putative STAND/prion-like couples show a diversity of effector, NOD, repeat domains and PFDs. These systems involve at least five different effector domains (HeLo, sesA, sesB, Goodbye, NAD and UPD-Phosphorylase), two types of NOD domains (NB-ARC and NACHT), four types of superstructure forming repeats (WD, ANK, TPR, HEAT repeats). Finally, we distinguish three types of putative PFDs. Apparently, these different building blocks can be assorted in various combinations (Figure 9).

## Discussion

Herein based on a series of *in silico* analyses we find that the NWD2 STAND protein encoded by the gene immediately adjacent to HET-S displays an N-terminal extension that is homologous to the HET-s PFD elementary structural motif and that these features are evolutionarily conserved in a range of fungal species. Based on the APAF-1 paradigm, we propose that the NWD2 protein can undergo ligand-induced oligomerization and that this oligomerisation brings the N-terminal PFD-like extensions into close proximity and allow their cooperative folding into the HET-s-like β-solenoid structure. This amyloid-fold would would then template HET-S into the β-solenoid fold and induce its toxicity. We thus envision the existence of two modes of activation of the HET-S HeLo domain, either by interaction with the prion form of HET-s (as occurs during incompatibility) or by interaction with the oligomeric form of NWD2.

Further sequence analyses suggest that a putative prion model of *N. haematococca* could also be regulated in the same way by a STAND protein. Our model, derived from fungal genome analyses, now awaits direct validation. Preliminary experimental data indicate that a NWD2 N-terminal peptide can form infectious amyloids and that NWD2 can induce [Het-s] prion formation *in vivo* thus providing some support for key aspects of the model (Asen Daskalov, unpublished results).

### On the Possible Biological Function of NWD2/HET-S System Pair in Innate Immunity

It is an established (and yet under recognized) fact that fungi are like any other organism under the threat of pathogens [59], [60]. Innate immunity in metazoans and plants is controlled by a repertoire of pathogen recognition receptors (PRRs) which correspond to STAND proteins; the NBS-LRR receptors in plants and the NOD-like receptors in animals cells [61]. We have proposed that the NWD-family and related fungal STAND proteins could represent fungal intracellular pathogen recognition receptors [62] and that the function of these proteins is to induce an immune inflammatory reaction in response to pathogen attack. Incompatibility could in fact be a by-product of the pathogen driven divergence in these genes whose primary function resides in innate immunity [62]; a kind of auto-immune condition. The use of STAND proteins as PRRs would then represents a strategy of innate immunity shared by fungi, animals and plants. As would be expected for molecules with a PRR function, the *nwd* gene family (including *nwd2*) is subject to positive Darwinian selection and capable of very rapid diversification [38], [63]. Most of these fungal STAND proteins, to which we attribute a PRR function, have the typical three domain organisation of PRRs with a N-terminal effector domain, a central NOD domain and a C-terminal repeated ligand-recognition domain (Figure 1D). The function of the effector domains would be, as in the case of animal and plant PRRs, to respond to the pathogen attack either by killing some of the host cells to prevent further invasion of the pathogen, by activating various defense responses or by directly targeting the pathogen. A number of effector domains of fungal STAND protein are annotated in as lytic enzymes (lipases, phosphorylases, proteases...), other bear cell death inducing domains like the HET or the HeLo domain. Among this arsenal of STAND proteins, we now reveal the existence of a subclass (exemplified by NWD2/HET-S) in which the effector domain is not directly associated with the NOD/recognition moiety but found *in trans* as a distinct proteins (encoded by a tighly linked gene). Communication between the NOD/recognition and effector modules appears to be based on transmission of the amyloid prion fold. In that model, NWD2 senses a pathogen-derived non-self ligand and oligomerizes, this oligomerization brings about the β-solenoid template which then converts the PFD-like region of HET-S into the same β-solenoid fold which ultimately leads to refolding and activation of the HeLo toxicity domain. By extension, we propose that the other STAND/effector domain pair identified in this study might have related functions in host defense. Remarkably, the involvement of prion-like aggregates in an antiviral innate immune response in mammals has recently been reported [64].

The nature of cognate NWD2 ligand is presently unknown but based on the extreme sequence homology between NWD2 and other HNWD proteins such as HET-E and HET-D [38], it is possible that these proteins might share the same ligand, specifically the glycolipid transfer protein encoded by the *het-c* gene.

### Implications for [Het-s]/HET-S Incompatibility

The description of NWD2 as an additional player in the [Het-s]/HET-S system, forces us to reconsider our understanding of the [Het-s]/HET-S system. In the model proposed here, the toxicity induced by activation of the HeLo toxicity domain of HET-S represents a host defense reaction. *het-s* strains in turn would be deficient in this activity because of the *het-s* HeLo domain fails to exert toxicity upon transconformation of the PFD region. In this model, the *het-s* allele thus represents a mutant allele having lost the function of the wild-type HET-S allele, namely the ability to induce cell death. In wild isolates of the *het-s* genotype, the *nwd2* gene is inactivated by insertion of a transposon [28], [58]. This correlated loss of NWD2 and HET-S activity might be explained by the fact that inactivation of either *nwd2* (by the insertion of the transposon) or *het-S* (by a point mutation in position 33 of the HeLo domain) renders the other interaction partner orphan so that no selective pressure remains to maintain gene function. At present, it cannot be resolved whether *nwd2* inactivation by the transposon insertion predated mutation of *het-S* or if these events occurred in the reverse order. Consistent with this hypothesis viewing the HET-s allele as a mutant form of HET-S, is the fact that based on sequence analysis of amino acid position 33 (which defines *het-s/het-S* allele specificity), HET-s homologs in other fungi are predicted to be of the HET-S and not HET-s type.

The [Het-s]/HET-S incompatibility system should now be viewed as a derivation, an evolutionary by-product of the NWD2/HET-S complex. The inactivation of the HET-s HeLo domain unleashed the [Het-s] prion since HET-s, in contrast to HET-S, can propagate as a harmless prion. Two hypotheses then emerge on the biological significance of [Het-s]/HET-S incompatibility. In the first hypothesis, *het-s* is simply an inactive form of *het-S* and *het-s* strain would present a deficit in host defense abilities. It should be recalled here that the *het-s* allele behaves as a meiotic drive element which skews mendelian segregation ratio in its favour in *het-s* x *het-S* sexual crosses [65]. This selfish genetic behaviour of *het-s* can explain the ongoing presence of *het-s* alleles in modern day populations even if the *het-s* allele is detrimental. The full implication of this first hypothesis is that the [Het-s] prion represents a -genetic and infectious- fungal disease. Alternatively, it can still be envisioned that a selective advantage is associated to [Het-s]/HET-S incompatibility as is classically proposed [66]. In that second hypothesis, *het-S* would represent a gene involved in pathogen defense that doubles as a heterokaryon incompatibility gene, while *het-s* (or better said the [Het-s] prion) solely functions in heterokaryon incompatibility. In that second hypothesis, [Het-s] is an adaptive prion.

### Transmission of an Amyloid Fold as an Integral Part of a Signal Transduction Process

It is clear today that amyloids are not strictly associated to pathologies and that some amyloids have functional roles [67], [68]. For instances, the curli fibrils of *E. coli* bacteria are amyloids. Our observations introduce a novel view on the biological significance of the amyloid fold transmission process as an integral part of cell fate determining signal transduction pathways. Several advantages of this mode of protein activation can be envisioned. In this model of propagation of the amyloid fold, the effector is turned into an inducer thus allowing for amplification of the signal, its propagation to adjacent cells and even hysteresis because the propagation of the response can become independent of the initial signal. In addition, when compared to the normal STAND signal transduction mechanisms (in which the effector module and the sensor module are carried on the same protein), the amyloid fold transmission mechanism allows a single STAND protein to activate simultaneously different effector domains and also to uncouple the expression level of the sensor/inducer (STAND) and effector moieties. The systems that we consider here are proposed to have their biological function in the inflammatory response to pathogenic non-self [62], these properties (amplification and propagation of the signal, hysteresis) might be particularly valuable in the context of an immune response so that the inflammatory response to a pathogen attack is rapidly propagated from the infected cell to the surrounding cells (filamentous fungi are syncytial organisms). The existence of positive feedback loops is very common in biological control systems [69]. Generally positive feedback loops and signal amplification systems involve chemical reactions (proteolysis, phosphorylation, gene induction…) but in the present system, positive feedback and amplification would rely solely on conformational modifications. Propagation of an amyloid fold might represent one of the simplest means to entail a biological control system with signal amplification, bistability and hysteresis.

Prion formation in yeast has been envisioned as an epigenetic switch of protein activity, a mechanism allowing for the establishment of a protein-based molecular memory [10]. The model we propose here is related to that concept but does nevertheless differ in two aspects. First, here the amyloid switch is used for protein activation rather than inactivation as seen with yeast prion models. Then, here the protein-based information is transmitted from one protein species to another, it is not restricted to an auto-inactivation mechanism by which a protein modifies more of itself. It has been shown in yeast, that cross-talk between distinct prion proteins species occurs; for instance the [*PIN* ^+^] prion is necessary the formation of [*PSI* ^+^] [70]. But whether this type of prion cross-induction mechanism is of adaptive value remains unclear and there is no evidence for primary sequence conservation between the PFDs of the corresponding proteins.

### Comparison of the PFD and PFD-like Motifs

We identify three distinct PFD-like motifs in the different STAND/effector domain pairs: the HET-s motif, PP-motif and the σ-motif. The total length of these conserved regions is relatively similar, 21 amino acids for the elementary HET-s motif, 17/18 amino acids for the PP-motif and 25–27 amino acids for the σ-motif. It is naturally premature to make inferences about the structural organization of these newly identified motifs. Yet, we note that all three motifs show sequence features reported in other amyloid forming domains. In particular, the motifs show conservation of G, N/Q and aromatic residues and are relatively poor in charged residues. This biased amino acid composition as been noted for yeast prion forming domains, for instance the Sup35 PFDs from various species are also rich in N, Q, Y and G and rarely contain charged residues [71]. The prevalence and functional importance of aromatic and N/Q residues in yeast prion forming domains and other amyloidogenic stretches is documented. N/Q residues allow formation of H-bonded axial “ladders” in amyloid fibrils and also lead to the formation of so-called steric-zippers [72], [73], [74], [75]. Aromatic residues allow stacking of π-bonds of the aromatic rings in the adjacent β-strands [76]. Finally, the conservation of the G residues in all three motifs might be understood in the context of formation of β-arcades, which structural elements connecting short adjacent β-strands which are very common in various amyloids [77]. The repeated structure of the σ-motif is also reminiscent of the oligopeptide repeats found in the Sup35 PFD [78] and palindromes as found in the PP-motif have been used to design peptide models for amyloid formation [79]. A first indication that at least the the σ-motif is also amyloidogenic is the observation that a synthetic peptide of the *N. haematococca het-eN* N-terminus corresponding to this σ-motif forms amyloid fibrils *in vitro* (Frédérique Ness, unpublished results).

### Evolutionary Aspects

We identify a total of 6 domains (HeLo, sesA, sesB, Goodbye, UDP-Phosphorylase and NAD) that could be regulated by the STAND/effector protein interaction we propose here. In all cases, STAND proteins in which these domains are found directly associated to the NOD/repeat moiety are also found in fungal genomes. It thus appears that two mechanistic solutions have been retained in the STAND signal transduction process. The effector domain can alternatively be located on the STAND protein (*in cis* mechanism) or communication between the STAND and effector involves transmission of the amyloid fold (*in trans* mechanism). Two evolutionary scenari can be envisioned to explain the existence of the two component organisation. First, the *in cis* organisation might be ancestral and the two component organisation appeared secondarily as a result of gene splitting event. Alternatively, the split organisation might be ancestral and a gene fusion event later generated the *in cis* organisation. At any rate, the co-existence of these two gene architectures in fungi suggest that both organisations posses intrinsic advantages.

What is the reason behind the genomic association of the *het-S* and *nwd2* and other STAND/effector protein couples? This clustering might be a simple consequence of the evolutionary history leading the formation of these systems. In other words, if *het-S* and *nwd2* have been formed by a gene splitting event, one might expect to find them as collinear and adjacent gene. This hypothesis however fails to explain that gene clustering is evolutionarily conserved while relative gene orientation is not. This hypothesis similarly cannot explain clustering of several distinct genes encoding different effector domains with the STAND encoding gene. Rather it appears that the tight genetic linkage between the two (or three) components is actually selected, it is as if the genes form a functional unit which is inherited as such. We find that PFD-like extensions apparently co-evolve within a gene cluster. This apparent concerted evolution of the effector and STAND protein extensions can be taken as a strong indication of the existence of a functional interaction between the effector domain and the STAND protein.

The mode of signal transduction that we propose here appears widespread both in terms of species distribution and the number of domains involved. We found STAND/effector domain pairs in at least 14 different pezizomycota species largely distributed in the fungal phylogenetic tree. Then, as mentioned above, we find that in addition to the HeLo toxicity domain, 5 other domains could be regulated in a similar manner. We distinguish three types of PFD-like domains. The σ-motif can be found associated to a variety of effector domains, in contrast the HET-s PFD is only found associated to the HeLo domain. In turn, certain effector domains like the HeLo and sesB domains can be found associated to different PFD-like motifs. Finally, the PFD-like extension are found associated to NOD domains belonging to two different clades of NOD domains (the NACHT and NB-ARC clade) and a variety of super-structure forming repeats. If our model is correct, it is clear that the proposed mechanism is not an isolated oddity concerning only the particular case of *P. anserina* NWD2/HET-S pair. At least in the fungal kingdom it is of wide occurrence. The diversity of effector domain and NOD domain types indicates that the generation of these systems (by gene fusion or fission) is the result of multiple reoccurring evolutionary events. It is as if the PFDs (putative or confirmed) behave as portable units that can be assorted with a variety of effector and NOD domains. Early yeast prion studies have shown that PFDs can behave experimentally as portable units [80]. The observations reported here suggest that these transfers of prion domains from one protein to another are indeed occurring in nature.

It will be of interest to determine whether similar systems exist in the genomes of organisms from other branches or if the presented mechanism is restricted to the fungal kingdom. If this is so, this might be related to the synthicial organisation of filamentous fungi in which the phenomenon of amyloid propagation could be exquisitely exploited for adaptive ends.

### Conclusions

Our observations identify NWD2 as a functional partner of the HET-S of *P. anserina*. More generally, we propose the existence of a common and evolutionary conserved functional interplay of STAND protein with effector domains in fungi ensured by transmission of amyloid folds.

## Methods

### Sequence Analyses

Fungal protein sequences were retrieve from ncbi (http://www.ncbi.nlm.nih.gov/) and the Broad Institute Fungal Genome Initiative web interface (https://www.broadinstitute.org/scientific-community/science/projects/fungal-genome-initiative/fungal-genome-initiative) in BLASTP and PSI-BLAST searches with default settings. Sequences alignment were performed with ClustalW2 (http://www.ebi.ac.uk/Tools/msa/clustalw2/) and formatted using the BOXSHADE server (http://www.ch.embnet.org/software/BOX_form.html). Consensus sequence and motif searches were performed using the MEME suite (http://meme.sdsc.edu/meme/intro.html). All sequence described and analyzed in the present study have been grouped in Table S1. Phylogenetic analyses were carried out in MEGA5.

### Homology Modeling

Homology modeling was performed using the Geno3D server (http://geno3d-pbil.ibcp.fr/cgi-bin/geno3d_automat.pl?page=/GENO3D/geno3d_home.html). The template for modeling was HET-s(218–289) structure (pdb: 2RNM, chains A, B, C, D). The results of the modelling are presented as a superposition of the 10 models calculated by Geno3D. For homology modeling, the chimeric sequences have been generated by replacing the r1 and r2 regions of the HET-s(218–289) with the 21 amino acid sequence of the STAND proteins.

## Supporting Information

Table S1 **Sequence described and analyzed in the present study.**
(XLS)
